# Metallotherapy

**Published:** 1877-08

**Authors:** 


### ARTICLE VI.

### *Metallotherapy.*

An interesting report has just been presented to the Societe de Biologie of Paris. In August 1876, M. Burq obtained the appointment of a committee, consisting of MM. Charcot, Luys, and Dumontpallier (reporter,) to investigate the effects of the application of various metals to the skin.

M. Burq has maintained for a long time (since 1849) that in certain affections of general and special sensation, a return of sensibility may be brought about by the cutaneous application of certain metals. The same metal does not equally affect all patients; in fact, there exists an idiosyncrasy towards this or that metal. The particular metal is easily ascertained by applying in succession for several minutes a plate or plates of gold, zinc, copper, or iron, to the affected parts. M. Burq has also maintained, in the face of much incredulity, that the affections of sensibility stand in direct relation to some general morbid condition, and that from the external application an indication is obtained for the internal administration of the same metal. This is the basis of his system of *Metallotherapie.*

The commission have not occupied themselves with M. Burq's metallotherapeutic speculations, but have confined their attention to the facts as the external application of the metals. The experiments of the committee were begun in November of last year in the Salpetriere, and have frequently been the cause of much astonishment to visitors to *clinique* of M. Charcot. The patients experimented on have been chiefly those affected with hysterical hemianæsthesia or hystero-epilepsy. In these cases, both superficial and deep common sensation are abolished unilaterally to such an extent, that not the slightest reaction is exhibited, even when long needles are thrust through and through parts usually the most sensitive. In fact, as M. Dumontpallier in his report says, the procedure by which the fact of an-

æsthesia was demonstrated "would have been barbarous, had it not been practised on parts devoid of sensibility." Taste and smell are likewise abolished on the same side. Colocynth may be placed on the side of the tongue, and the most disagreeable odours held to the nostril, without exciting the slightest appearance of sensation or reaction. As regards sight and hearing, loss on the anæsthetic side is not usually complete but considerable, there being no signs of organic disease in the retina or ear. The temperature is also found to be lower on the anæsthetic side; and the muscular power, as tested by the dynamometer, considerably impaired. The committee have found, in accordance with M. Burq's statements, that there exists in different patients a special *aptitude metallique;* that is to say, that sensibility returns under the influence of a certain metal. This is determined by bending on the arm or leg, or anæsthetic part, plates of gold, copper, zinc, etc. After from ten to fifteen minutes, it is found that if the metal employed be the proper one, sensation begins to return at the point of application. The patient feels a sensation of heat or numbness; the part becomes red; and pricking with a needle causes great pain at the level of the bracelet and for some distance above and below it. At a given distance, well marked dysæsthesia, or perverted sensibility, becomes established.

When the metal is applied to the forearm, the sensibility gradually extends to the hand, and the temperature rises above that of the other side, as determined by a thermometer fixed in each hand. At the same time the muscular power rises very considerably above that which existed before the commencement of the experiment. Curiously, also, needle punctures now bleed freely; whereas in the anæsthetic condition scarcely a drop of blood showed itself even when the needle was thrust through the limb. What is true of common sensibility is also true of the special senses. By the application of the metal at or near the organs of special sense, a more or less complete restoration is effected.

The effect, however, is very transitory, and next day the anæsthesia is found to have returned. The patients generally complain of great fatigue, headache, and a tendency to sleep after each experiment. But perhaps the most remarkable phenomenon of all is that the return of sensibility under the influence of the metallic plates, is effected at the expense of the sensibility of the sound side. To this phenomenon the committee give the name *transfert de la sensibilite.* This takes place in a symmetrical manner. Thus, if the sensibility be restored to the arm by the application of the metal, it is found that the corresponding part of the sound arm has lost its normal sensibility. The same is true of the organs of special sense, and of temperature and muscular power. It would seem, therefore, that there is no real gain, but that a *plus* on the one side is accompanied by a *minus* on the other.

The committee have not yet ascertained the effect of the simultaneous application of the metals on both sides of the body. But experiments have been made on two cases of hemianæsthesia and hemichorea on the right side consequent on an attack of hemiplegia from organic cerebral mischief. This patient was subjected to experimentation with differant metals—zinc, copper, gold and iron. The result was negative as regards zinc, copper, and gold; but iron, applied to the thigh, arm and forehead, caused return of sensibility after twenty minutes' application. Sensibility gradually extended over the whole of the right side, and the hemichorea was sensibly diminished. Plates of iron applied over the nose and tongue also restored sensation there. Similar results ensued in another case on the application of gold. It is further remarkable that in these two cases the recovery has been permanent and not transitory as in the hysterical cases. M. Burq's statements having thus been fully confirmed, the committee next endeavoured to ascertain the *modus operandi* of the metallic plates. It having been suggested by several members of the society that the results were due to electrical action set up by contact of the metals with the skin, M. Regnard was entrusted with the experimental testing of the hypothesis. From Regnard's experiments with Du Bois Reymond's galvanometer, the fact was demonstrated that the contact of the metals with the skin did actually excite electrical currents, which varied with the metal employed. Thus the plates of gold caused a deflection of the needle of from two to twelve degrees, while similar plates of copper caused a deflection of from forty to forty-five degrees. This being established, it was rational to suppose that currents of like intensity furnished by a galvanic pile would produce effects analogous to those caused by the application of the metals. This also was experimentally verified, it being found that in those patients sensible to gold, a current of from two to twelve degrees caused a restoration of sensibility, and in those sensible to copper a current of from forty to forty-five degrees was necessary. A further interesting fact was made out in the course of these experiments, viz., that on varying the intensity of the currents, active and neutral points were found to exist in the galvanometic scale. Thus a patient sensitive to a current of 10 to 15 deg. ceased to be sensitive to a current of 45 to 90 deg., but again became sensitive to a current of 80 to 90 deg. The points of non-activity are termed by M. Regnard the *neutral points* of the scale.

What the explanation of these curious facts may be, is left undetermined by the committee.

The results of this investigation are so novel and so unexpected, that they cannot fail to stimulate further speculation and research. The committee compliment M. Burq on the favourable results of their labours, and recommend his memoir for competition for the Prix Ernest Godard.—*British Med. Journal.*

---

